# Antimicrobial therapeutic determinants of outcomes from septic shock among patients with cirrhosis

**DOI:** 10.1002/hep.25931

**Published:** 2012-12-04

**Authors:** Yaseen M Arabi, Saqib I Dara, Ziad Memish, Abdulmajeed Al Abdulkareem, Hani M Tamim, Nehad Al-Shirawi, Joseph E Parrillo, Peter Dodek, Stephen Lapinsky, Daniel Feinstein, Gordon Wood, Sandra Dial, Sergio Zanotti, Anand Kumar

**Affiliations:** 1 Intensive Care Department, King Abdulaziz Medical CityRiyadh, Saudi Arabia; 5 Department of Hepatobiliary Surgery and Liver Transplantation, King Saud bin Abdulaziz University for Health SciencesKing Abdulaziz Medical City, Riyadh, Saudi Arabia; 6 Department of Epidemiology and Biostatistics, College of Medicine, King Saud bin Abdulaziz University for Health Sciences, King Abdulaziz Medical CityRiyadh, Saudi Arabia; 2 Department of Respiratory Services, King Abdulaziz Medical CityRiyadh, Saudi Arabia; 3 Department of Infectious Diseases, Preventive Medicine Directorate, Ministry of HealthRiyadh, Saudi Arabia; 4 College of Medicine, Alfaisal UniversityRiyadh, Saudi Arabia; 7 Department of Medicine, Cooper Medical School of Rowan UniversityCamden, NJ; 8 St. Paul's Hospital, University of British ColumbiaVancouver, BC, Canada; 9 Section of Critical Care Medicine, Mount Sinai Hospital, University of TorontoToronto, ON Canada; 10 Moses H. Cone Memorial HospitalGreensboro, NC; 11 Royal Jubilee Hospital/Victoria General Hospital, University of British ColumbiaVictoria, BC, Canada; 12 Section of Pulmonary Medicine, McGill UniversityMontreal, QC, Canada; 13 Cooper Medical School of Rowan UniversityCamden, NJ; 14 Department of Medical Microbiology and Pharmacology/Therapeutics, Section of Critical Care Medicine and Section of Infectious Diseases, Health Sciences Center and St. Boniface Hospital, University of ManitobaWinnipeg, MB, Canada; 15 Division of Cardiovascular Diseases and Critical Care Medicine, Cooper Medical School of Rowan UniversityCamden, NJ

## Abstract

It is unclear whether practice-related aspects of antimicrobial therapy contribute to the high mortality from septic shock among patients with cirrhosis. We examined the relationship between aspects of initial empiric antimicrobial therapy and mortality in patients with cirrhosis and septic shock. This was a nested cohort study within a large retrospective database of septic shock from 28 medical centers in Canada, the United States, and Saudi Arabia by the Cooperative Antimicrobial Therapy of Septic Shock Database Research Group between 1996 and 2008. We examined the impact of initial empiric antimicrobial therapeutic variables on the hospital mortality of patients with cirrhosis and septic shock. Among 635 patients with cirrhosis and septic shock, the hospital mortality was 75.6%. Inappropriate initial empiric antimicrobial therapy was administered in 155 (24.4%) patients. The median time to appropriate antimicrobial administration was 7.3 hours (interquartile range, 3.2-18.3 hours). The use of inappropriate initial antimicrobials was associated with increased mortality (adjusted odds ratio [aOR], 9.5; 95% confidence interval [CI], 4.3-20.7], as was the delay in appropriate antimicrobials (aOR for each 1 hour increase, 1.1; 95% CI, 1.1-1.2). Among patients with eligible bacterial septic shock, a single rather than two or more appropriate antimicrobials was used in 226 (72.9%) patients and was also associated with higher mortality (aOR, 1.8; 95% CI, 1.0-3.3). These findings were consistent across various clinically relevant subgroups. *Conclusion:* In patients with cirrhosis and septic shock, inappropriate and delayed appropriate initial empiric antimicrobial therapy is associated with increased mortality. Monotherapy of bacterial septic shock is also associated with increased mortality. The process of selection and implementation of empiric antimicrobial therapy in this high-risk group should be restructured. (Hepatology 2012;56:2305–2315)

Chronic liver disease and cirrhosis result in an estimated 800,000 deaths each year worldwide.^1^ In the United States alone, it is the ninth leading cause of death, with about 30,000 deaths each year.^2^ An additional 30 million Americans have chronic liver impairment.^3^ Hospitalizations of these patients are frequent and substantial proportions of these admissions include stays in the intensive care unit (ICU).^4–6^ The estimated number of ICU admissions related to cirrhosis in the United States alone is in excess of 26,000 per year with an estimated cost of $3 billion.^7^

A major cause of ICU admission among patients with cirrhosis is sepsis.^5^, ^8–10^ The incidence of sepsis is estimated to be at least 30%-50% of hospital admissions in this group.^11^, ^12^ Cirrhosis-associated septic shock stands out in terms of presentation, outcome,^13^ and therapeutic options.^14^ One of the key questions is whether modifiable practice-related factors contribute to the poor outcome in this group of patients. Limited data are available about the appropriate application of the newer options that have emerged in the management of sepsis over the last decade^15–17^ in this high-risk group,^18^ as patients with cirrhosis-related septic shock are often excluded from clinical trials. In addition to this paucity of evidence-based information,^19^ the Surviving Sepsis Campaign guidelines do not provide a clear direction for this group of patients.^20^

In a heterogeneous patient population with septic shock, the early initiation of appropriate antimicrobials and combination antibiotics (for bacterial septic shock) is associated with higher survival rate.^21–23^ However, few data exist on the use of antibiotics and outcome from septic shock among patients with cirrhosis.^11^ Such lack of information may adversely affect decision-making about patient management and prognostication.

We conducted this study to examine the relationship between the aspects of early, initial empiric antimicrobial therapy and hospital mortality in patients with cirrhosis and septic shock.

## Patients and Methods

### Patients and Setting

This was a nested cohort study within a retrospective database on septic shock conducted in 28 medical centers in Canada, the United States, and Saudi Arabia by the Cooperative Antimicrobial Therapy of Septic Shock (CATSS) Database Research Group between 1996 and 2008. The details of the setting have been described elsewhere.^22^ Data were extracted for all adult patients with cirrhosis (biopsy-proven cirrhosis, documented variceal hemorrhage or portal hypertension, hepatic ascites, or encephalopathy). Approval was obtained from the Institutional Review Boards of all participating institutions.

### Measurements

We collected baseline patient characteristics including demographics and comorbid conditions. The following data were obtained on day 1 of septic shock: serum lactate, bilirubin, creatinine, and bicarbonate levels, platelet count, international normalized ratio and white blood cell (WBC) count, and acute physiology and chronic health evaluation (APACHE) II^24^ score. We calculated the model for end-stage liver disease (MELD) score on day 1 as described previously.^25^

### Outcomes

Hospital mortality was considered the primary outcome variable. Secondary outcomes were ICU mortality and hospital and ICU length of stay.

### Definitions

Septic shock was defined using the 1992 American College of Chest Physicians/Society of Critical Care Medicine guidelines.^26^ As per that definition, case patients were required to have documented or suspected infection, persistent hypotension requiring therapy with vasopressors, and two of the following four elements: (1) a heart rate of >90 beats/minute; (2) a respiratory rate of >20 breaths/minute or arterial partial pressure of carbon dioxide (PaCO_2_) of <32 mm Hg; (3) a core temperature of <36°C or >38°C; and (4) a WBC count of <4,000/μL or >12,000/μL or bands >10%. An episode of hypotension was considered to represent the initial onset of septic shock when hypotension persisted from onset despite fluid (2 L of saline or equivalent) administration (persistent hypotension) or hypotension was only transiently improved (hypotension resolution for <1 hour) with fluid resuscitation (recurrent hypotension).^21^ Predetermined rules were used to define documented and suspected infections and to assign significance to clinical isolates (see Supporting Information). For patients with multiple isolated organisms, we identified the primary organism that was likely responsible for the infection. We documented the following multidrug-resistant organisms: Methicillin-resistant *Staphylococcus aureus*, carbapenem-resistant gram-negative organisms, vancomycin-resistant enterococci and extended-spectrum beta-lactamase–producing Enterobacteriaceae. Nosocomial infection-related septic shock was defined as septic shock caused by any infection developing >48 hours after hospital admission. Cases not meeting this definition were considered to be septic shock associated with community-acquired infections. We used the term “immunocompromised patients” for a subgroup of patients who had one of the following comorbidities: acquired immunodeficiency syndrome, acute or chronic lymphoma, acute or chronic leukemia/multiple myeloma, metastatic solid cancer, immunosuppressive chemotherapy, or long-term steroid therapy (>10 mg prednisone equivalent daily). Other patients were labeled as “non-immunocompromised.”

Predetermined rules used to assess the appropriateness and delays of initial antimicrobial therapy^21–23^ are summarized below and detailed in the Supporting Information. For culture-positive septic shock, initial antimicrobial therapy was considered appropriate if an antimicrobial with *in vitro* activity appropriate for the isolated pathogen or pathogens was the first new antimicrobial agent given after the onset of recurrent or persistent hypotension or was initiated within 6 hours of the administration of the first new antimicrobial agent. Otherwise, the initial therapy was considered inappropriate.^22^ For culture-negative septic shock, initial therapy was considered appropriate when an antimicrobial agent consistent with broadly accepted norms for empiric management of the typical pathogens for the clinical syndrome was the new antimicrobial agent given after the onset of recurrent or persistent hypotension or was initiated within 6 hours of administration of the first new antimicrobial agent.^22^ At each participating institution, infectious disease physicians/microbiologists were consulted to account for the local community and nosocomial flora when considering appropriateness of empiric therapy during the period covered by data collection. Otherwise, appropriate empiric therapy of culture-negative infections leading to septic shock was based on the recommendations listed in the “Clinical Approach to Initial Choice of Antimicrobial Therapy” in the Sanford Guide to Antimicrobial Therapy 2004 (34th edition).^27^ For the purposes of this study, antibiotic monotherapy was defined as the administration of any single, appropriate, intravenous, preferably bactericidal antibiotic at any point after the onset of recurrent or persistent hypotension. Combination therapy was defined as the concomitant use of two or more antibiotics of different mechanistic classes with activity for the isolated or suspected pathogens. The second agent had to be started within 24 hours of the first antibiotic or within 24 hours of the onset of hypotension (if the first agent was initiated before hypotension was documented).^23^ Patients with septic shock due to yeast, anaerobic, or mycobacterial infection were excluded from this analysis of single versus combined antibiotics.

### Statistical Analysis

Continuous variables are reported as the mean ± SD and median (interquartile range) as appropriate. Categorical variables are reported as numbers and percentages. The Student *t* test, Mann-Whitney U test, chi-square test, and Fisher's exact test for comparison between groups were used, as appropriate.

To study the association between appropriateness, timing, and combination of antimicrobial/antibiotic therapy and hospital mortality (dependent variable), forward step-wise logistic regression analyses were performed. The following independent variables were included based on their significance in the univariate analysis: APACHE II score, MELD score, immunocompromised (versus non-immunocompromised), bacteremia (versus no bacteremia), community-acquired (versus nosocomial), and culture-positive (versus culture-negative).

To determine the predictors of inappropriate antimicrobial and single antibiotic therapy, we performed forward stepwise logistic regression analyses. In the first analysis, inappropriate antimicrobial therapy was the dependent variable, and the independent variables were age, sex, BMI, APACHE II score, MELD score, serum lactate, bilirubin, creatinine and bicarbonate levels, platelet count, international normalized ratio, WBC count, heart rate, temperature, respiratory rate, blood pressure, community-acquired (versus nosocomial), organ failures, activated protein C, steroids, multidrug-resistant organisms, and comorbidities. A similar analysis was performed for single antibiotic therapy as the dependent variable.

A third analysis was performed to assess the predictors of delayed antimicrobial therapy using forward stepwise linear regression analysis with the same independent variables listed above.

We performed subgroup analyses for the following categories: septic shock with documented and suspected infection, culture-positive and culture-negative, bacteremia and no bacteremia, community-acquired and nosocomial infections, gram-positive and gram-negative infections, pneumonia, intra-abdominal infection, immunocompromised and non-immunocompromised, and country of origin (Canada, United States, and Saudi Arabia). To adjust for the impact of potential changes in practice over time, we divided the study time period into four quartiles and compared outcomes across the four periods. To examine the possibility of effect modification, we tested for interaction of the above-mentioned subgroups with the appropriateness, timing and combination of antimicrobial in the related multivariate models.

For all multivariate analyses, we checked for multicollinearity among covariates by evaluating the variation inflation factors. Missing data were handled using the mean and median imputation method.^28^ Results were reported as adjusted odds ratios (aOR) and 95% confidence intervals (CI). *P* < 0.05 was considered significant. SAS software (SAS Institute, Cary, NC) was used for statistical analyses.

## Results

Among the 8,670 patients with septic shock within the CATSS database, we identified 635 (7.3%) patients with cirrhosis (385 men [60.6%], 250 women [39.4%]). The mean age (± SD) was 55.5 ± 12.7 (Table 1). The first day mean APACHE II and MELD scores were 28.2 ± 8.2 and 26.7 ± 11.1, respectively. The frequencies of chronic comorbidities among the patient cohort are presented in Table 1. Nearly half of the patients suffered from chronic alcohol abuse. The most common sites of infection were lung (37%), intra-abdominal (35%), and primary bloodstream (7.9%) (Table 2). Positive cultures were obtained in 473 (74.5%) patients. The most common isolated pathogens were gram-negative (35.1%) followed by gram-positive (26.5%) and fungi (9.3%). Thirty-one (4.9%) patients had multidrug-resistant organisms (Table 3).

**Table 1:** Baseline Characteristics of Patients With Cirrhosis and Septic Shock

Characteristic	Value
Age, years	55.5 ± 12.7
Sex, men/women	385 (60.6)/250 (39.4)
BMI, kg/m^2^	27.8 ± 7.8
APACHE II score	28.2 ± 8.2
MELD score	26.7 ± 11.1
No. of organ failures on day 1	4.7 ± 1.7
Mechanical ventilation	471 (74.2)
Laboratory findings on day 1	
Lactate, mmol/L	6.4 ± 4.8
Bilirubin, μmol/L	142 ± 171
Creatinine, μmol/L	215 ± 167
Bicarbonate, mmol/L	17.3 ± 6.2
Platelet count, ×10^9^/L	128 ± 119
INR	2.4 ± 1.5
WBC count, ×10^9^/L	15.7 ± 12.4
Vital signs	
Heart rate, beats/minute	114 ± 28
Respiratory rate, beats/minute	27 ± 9
Temperature, °C	36.9 ± 1.8
Mean arterial pressure, mm Hg	56 ± 14
Vasopressor use	635 (100)
Renal replacement therapy	56 (8.8)
Activated protein C	9 (1.4)
Steroids	192 (30.2)
Comorbidities	
AIDS	19 (3.0)
Acute or chronic lymphoma	10 (1.6)
Acute or chronic leukemia/multiple myeloma	9 (1.4)
Metastatic solid cancer	26 (4.1)
Immunosuppressive chemotherapy or long-term steroid therapy (>10 mg prednisone equivalent daily)	54 (8.5)
Neutropenia (>500 cells/L)	12 (1.9)
New York Heart Association class IV heart failure	34 (5.4)
COPD (requiring medication or oxygen)	27 (4.3)
Chronic renal failure*	74 (11.7)
Chronic dialysis dependence	31 (4.9)
Diabetes mellitus (medication-dependent)	100 (15.8)
Diabetes mellitus (insulin-dependent)	54 (8.5)
Alcohol abuse	278 (43.8)
Elective surgery	64 (10.1)
Emergency surgery/trauma	33 (5.2)
Bacteremia/fungemia	245 (38.6)
Community-acquired infection	357 (56.2)
Nosocomial infection	278 (43.8)

Continuous variables are expressed as the mean ± SD. Categorical variables are expressed as no. (%).

Abbreviations: AIDS, acquired immune deficiency syndrome; BMI, body mass index; COPD, chronic obstructive pulmonary disease; INR, international normalized ratio. All percentages are out of the total number of patients in the cohort (n = 635).

* Serum creatinine >1.5 the upper limit of normal.

**Table 2:** Clinical Sites of Infection Among the Patient Cohort

Site of infection	Value
Lung	235 (37.0)
Pneumonia	228 (35.9)
Empyema	7 (1.1)
Intra-abdominal	222 (35.0)
Intra-abdominal abscess	14 (2.2)
Ascending cholangitis	17 (2.7)
Cholecystitis	8 (1.3)
Ischemic bowel/bowel infarction	25 (3.9)
Bowel perforation/peritonitis	23 (3.6)
Spontaneous bacterial peritonitis	112 (17.6)
Clostridium difficile enterocolitis/toxic megacolon	7 (1.1)
Others	16 (2.5)
Skin and soft tissue	29 (4.6)
Cellulitis	6 (0.9)
Necrotizing soft tissue infections	19 (3.0)
Others	4 (0.6)
Genitourinary	41 (6.5)
Intravascular catheter infection	18 (2.8)
Primary bloodstream (bacteremia/fungemia without identifiable source)	50 (7.9)
Systemically disseminated (including yeast and tuberculosis)	22 (3.5)
Septic arthritis	7 (1.1)

Data are expressed as no. (%). All percentages are out of the total number of patients in the cohort (n = 635). Clinical sites of infection were documented in 548 (86.3%) patients and suspected in 87 (13.7%) patients.

**Table 3:** Primary Organisms Among the Patient Cohort

Organism	Value
Gram-negative*	223 (35.1)
*Escherichia coli*	95 (15.0)
*Klebsiella* species	46 (7.2)
*Pseudomonas aeruginosa*	26 (4.1)
*Enterobacter* species	13 (2.0)
*Haemophilusinfluenzae*	12 (1.9)
*Acinetobacter* species	7 (1.1)
*Serratia* species	5 (0.8)
*Stenotrophomonas maltophilia*	6 (0.9)
Other gram-negative organisms	13 (2.0)
Gram-positive*	168 (26.5)
*Staphylococcus aureus*	74 (11.7)
*Streptococcus pneumoniae*	37 (5.8)
*Streptococcusfaecalis*	12 (1.9)
Group A *Streptococcus* species	8 (1.3)
Other β-hemolytic *Streptococcus* species	12 (1.9)
Viridans *Streptococcus* species	9 (1.4)
*Streptococcus faecium*	12 (1.9)
Other gram-positive organisms	4 (0.6)
Yeast/fungus	59 (9.3)
*Candida albicans*	40 (6.3)
*Candida glabrata*	8 (1.3)
*Candida tropicalis*	5 (0.8)
Other *Candida* species/yeast	6 (0.9)
Anaerobes	12 (1.9)
*Clostridium difficile*	7 (1.1)
*Bacteroides fragilis*	2 (0.3)
Other *Clostridium* species	1 (0.2)
Other anaerobes	2 (0.3)
Other organisms	11 (1.7)
Total culture-positive	473 (74.5)
Total culture-negative	162 (25.5)
Multidrug-resistant	31 (4.9)
Methicillin-resistant *Staphylococcus aureus*	17 (2.7)
Carbapenem-resistant gram-negative bacteria	8 (1.3)
Vancomycin-resistant *enterococci*	3 (0.5)
ESBL-producing Enterobacteriaceae	3 (0.5)

Data are expressed as no. (%). All percentages are out of the total number of patients in the cohort (n = 635).

Abbreviation: ESBL, extended-spectrum beta-lactamase.

* Includes multidrug-resistant organisms.

The ICU and hospital mortality rates were 61.6% and 75.6%, respectively. The mean ICU length of stay was 9.9 ± 11.5 days, and the mean hospital length of stay was 17.8 ± 25.2 days, respectively. Hospital mortality was similar over the four study period quartiles. Hospital nonsurvivors had higher APACHE II and MELD scores (Table 4) and were more likely to receive inappropriate initial empiric antimicrobials (30.6% versus 5.2%) and delayed appropriate empiric antimicrobial therapy (median (interquartile range)) (10.0 (4.9–23.8) versus 3.2 (1.3–6.8) hours) than survivors. Nonsurvivors with bacterial septic shock were also more likely to have been treated with empiric mono-antimicrobial therapy (77.0% versus 63.4%).

**Table 4:** Comparison of Patient Characteristics Between Hospital Survivors and Nonsurvivors Among the Patient Cohort

Variable	Hospital Survivors	Hospital Nonsurvivors	*P*
No. of patients	155	480	—
Age, years	54.5 ± 12.8	55.9 ± 12.7	0.22
Sex, men/women	103 (66.5)	282 (58.8)	0.09
BMI, kg/m^2^	27.8 ± 7.9	27.8 ± 7.8	0.96
APACHE II score	22.8 ± 6.5	29.9 ± 8.0	<0.0001
MELD score	22.2 ± 10.1	28.1 ± 11.0	<0.0001
Laboratory findings on day 1			
Lactate, mmol/L	5.5 ± 4.2	6.7 ± 5.0	0.47
Bilirubin, μmol/L	85 ± 117	160 ± 182	<0.0001
Creatinine, μmol/L	201 ± 177	220 ± 163	0.22
Bicarbonate, mmol/L	19.0 ± 6.0	16.6 ± 6.2	0.0006
Platelet count, ×10^9^/L	156 ± 143	119 ± 108	0.004
INR	2.0 ± 1.5	2.5 ± 1.5	0.0006
WBC count, ×10^9^/L	17.3 ± 12.8	15.2 ± 12.2	0.09
Vital signs			
Heart rate, beats/minute	114 ± 27	115 ± 29	0.73
Respiratory rate, beats/minute	25 ± 10	28 ± 9	0.009
Temperature, °C	37.4 ± 1.6	36.7 ± 1.9	0.0002
Mean arterial pressure, mm Hg	60 ± 16	55 ± 14	0.05
Activated protein C	2 (1.3)	7 (1.5)	1.00
Steroids	48 (31.0)	144 (30.0)	0.82
Inappropriate antimicrobials	8 (5.2)	147 (30.6)	<0.0001
Single appropriate antibiotic	59 (63.4)	167 (77.0)	0.01
Delay in effective antimicrobials, hours	3.2 (1.3–6.8)	10.0 (4(.9–23.8)	<0.0001
Comorbidities			
AIDS	1 (0.7)	18 (3.8)	0.06
Acute or chronic lymphoma	2 (1.3)	8 (1.7)	1.00
Acute or chronic leukemia/multiple myeloma	1 (0.7)	8 (1.7)	0.70
Metastatic solid cancer	5 (3.2)	21 (4.4)	0.53
Immunosuppressive chemotherapy or long-term steroid therapy (>10 mg prednisone equivalent daily)	9 (5.8)	45 (9.4)	0.17
Neutropenia (>500 cells/L)	1 (0.7)	11 (2.3)	0.31
New York Heart Association class IV heart failure	9 (5.8)	25 (5.2)	0.77
COPD (requiring medication or oxygen)	7 (4.5)	20 (4.2)	0.85
Chronic renal failure*	14 (9.0)	60 (12.5)	0.24
Chronic dialysis dependence	6 (3.9)	25 (5.2)	0.50
Diabetes mellitus (medication-dependent)	26 (16.8)	74 (15.4)	0.69
Diabetes mellitus (insulin-dependent)	13 (8.4)	41 (8.5)	0.95
Alcohol abuse	74 (47.7)	204 (42.5)	0.25
Elective surgery	12 (7.7)	52 (10.8)	0.27
Emergency surgery/trauma	8 (5.2)	25 (5.2)	0.98
Culture-positive	106 (68.4)	367 (76.5)	0.05
Bacteremia	57 (36.8)	188 (39.2)	0.59
Community-acquired infection	104 (67.1)	253 (52.7)	0.002
Nosocomial infection	51 (32.9)	227 (47.3)	0.002

Continuous variables are expressed as the mean ± SD or median and interquartile range. Categorical variables are expressed as no. (%).

Abbreviations: AIDS, acquired immune deficiency syndrome; BMI, body mass index; COPD, chronic obstructive pulmonary disease; INR, international normalized ratio.

* Serum creatinine >1.5 the upper limit of normal.

Of the 635 patients with cirrhosis and septic shock, inappropriate initial antimicrobial therapy was administered in 155 (24.4%) (Table 5). Forty-six (7.2%) patients never received appropriate antimicrobials before death. The median (interquartile range) time to administration of antimicrobials was 7.3 (3.2–18.3) hours after documentation of hypotension associated with septic shock. Two hundred twenty-six (72.9%) of the 310 patients with eligible bacterial septic shock who could potentially receive combination antibiotic therapy received a single antibiotic. There were no significant differences in these antibiotic-related variables across the three countries (see Supporting Information), except for a higher proportion of patients receiving combination therapy during the course of shock in United States hospitals (25.3% of eligible patients in Canada, 50% in the United States, 24.6% in Saudi Arabia; *P* = 0.03).

**Table 5:** Descriptive Analysis of Antimicrobial Determinants and Patient Outcomes

	Total	Mortality	P*
Appropriateness of initial antimicrobial therapy, no. (%)			
Inappropriate	155 (24.4)	147 (94.8)	<0.0001**
Culture-positive	128 (20.2)	120 (93.8)	
Culture-negative	27 (4.3)	27 (100.0)	
Appropriate	480 (75.6)	333 (69.4)	
Culture-positive	345 (54.3)	247 (71.6)	
Culture-negative	135 (21.3)	86 (63.7)	
Timing of first appropriate antibiotic, no. (%)			
Prior to hypotension onset	113 (17.8)	84 (74.3)	0.68
After hypotension onset	476 (75.0)	349 (73.3)	
Hours after hypotension, median (IQR)	7.3 (3.2–18.3)		
Appropriate antimicrobial therapy during the course of shock, no. (%)			
Never received appropriate antimicrobials	46 (7.2)	46 (100.0)	<0.0001
Received appropriate definitive therapy	589 (92.8)	434 (73.7)	
Potential candidates for combined antibiotic therapy	310 (48.8)		
Received single therapy	226 (72.9)	167 (73.9)	0.01
Received combination therapy	84 (27.1)	50 (59.5)	

Abbreviation: IQR, interquartile range.

* *P* values are for the comparisons of mortality.

** Comparison of mortality between inappropriate and appropriate initial antimicrobial therapy.

### Impact of Inappropriateness of Initial Antimicrobial Therapy on Hospital Mortality

**Fig 1:**
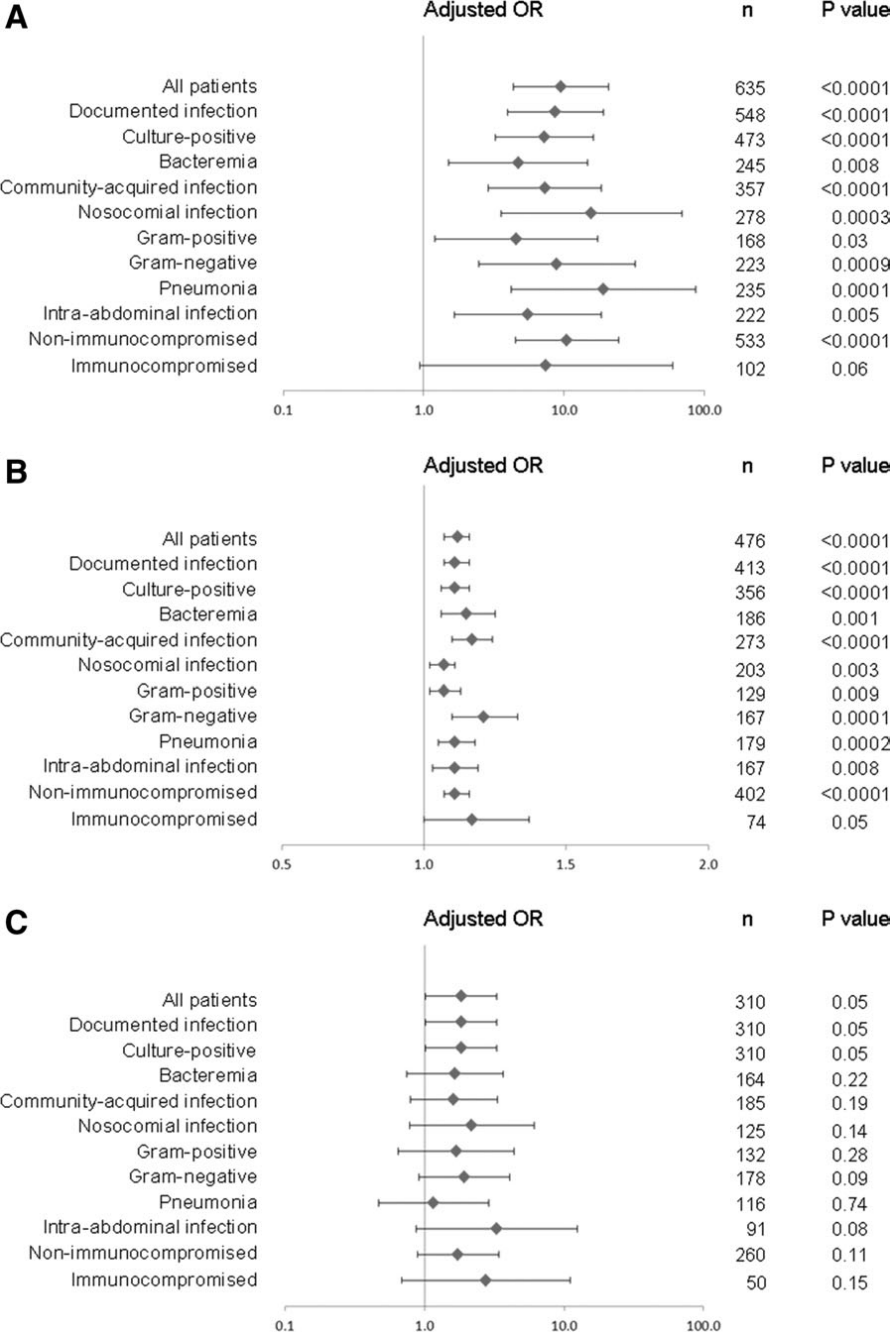
Association of inappropriate antimicrobial therapy (A), hours of delay in effective antimicrobial therapy (B), and use of single versus combined antimicrobial therapy (C) with hospital mortality across various subgroups of patients using multivariate analyses. The following independent variables were entered in the model: APACHE II score, MELD score, immunocompromised (versus non-immunocompromised), bacteremia (versus no bacteremia), community-acquired (versus nosocomial), and culture- positive (versus culture-negative). The results are shown as aOR and 95% CI on a logarithmic scale.

The use of inappropriate antimicrobials as initial therapy was associated with a significant increase in mortality (aOR, 9.5; 95% CI, 4.3–20.7) (Fig. 1A). Tests of interaction indicated that this finding was consistent in all tested subgroups of patients, whether with documented or suspected infections, culture-positive or culture-negative, bacteremia or no bacteremia, community-acquired or nosocomial infections, gram-positive or gram-negative infections, pneumonia, intra-abdominal infection, immunocompromised or non-immunocompromised, and across countries (Canada, United States, and Saudi Arabia) and the four study periods. Results of the interaction tests are presented in the Supporting Information. Figure 1A shows the aOR and 95% CI for selected subgroups.

### Impact of Timing of Initial Antimicrobial Therapy on Hospital Mortality

**Fig 2:**
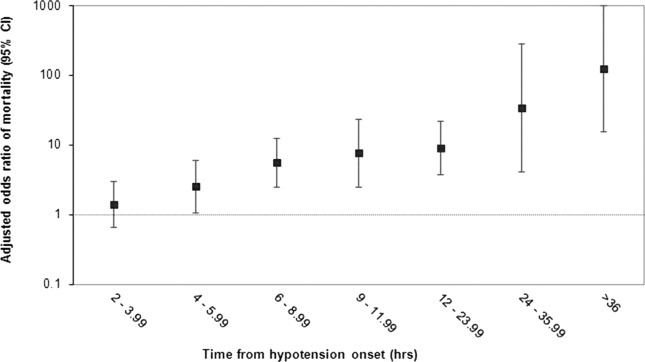
aOR and 95% CI of hospital mortality (on logarithmic scale) by the time from the onset of hypotension to the antimicrobial therapy in hours. Adjustments were made for the following independent variables: APACHE II score, MELD score, immunocompromised (versus non-immunocompromised), bacteremia (versus no bacteremia) community-acquired (versus nosocomial), and culture-positive (versus culture-negative).

The delay in use of appropriate antimicrobials was associated with a significant increase in mortality (aOR, 1.1; 95% CI, 1.1–1.2 per hour of delay) after onset of shock (Figs. 1B and 2). Tests of interaction indicated that this finding was also consistent in all tested subgroups of patients (see Supporting Information). Figure 1B shows the aOR and 95% CI for selected subgroups.

### Impact of Combination Antibiotic Therapy on Hospital Mortality

The use of a single antibiotic for bacterial septic shock was associated with a significant increase in mortality (aOR, 1.8; 95% CI, 1.0–3.3) (Fig. 1C). Tests of interaction indicated that this finding was also consistent in all tested subgroups of patients (see Supporting Information). Figure 1C shows the aOR and 95% CI for selected subgroups.

### Clinical, Laboratory, and Microbiological Predictors of Suboptimal Antimicrobial Therapy

We identified the multidrug-resistant organisms (aOR, 3.1; 95% CI, 1.5–6.4) as a predictor of inappropriate antimicrobial therapy. The following were the predictors of delay in initial antimicrobial therapy: patients who had a lower presenting temperature (*P* = 0.003), higher initial serum bicarbonate concentration (*P* = 0.02), nosocomial infections (*P* = 0.0009), and who were female (*P* = 0.05). We did not find any significant predictors of single versus combined antimicrobial therapy on multivariate analysis.

When appropriateness, timing, and combination of antimicrobials were compared according to the micro-organisms, fungal infections were found to be more likely associated with inappropriate and delayed antimicrobial therapy (*P* < 0.001 for both). We found the following variables to be significantly associated with the development of fungal infections: higher MELD score (*P* = 0.007), chronic renal failure (*P* = 0.009), higher bilirubin (*P* = 0.002), lower heart rate (*P* = 0.03), and lower body mass index (*P* = 0.05) on univariate analysis.

## Discussion

In this study, the hospital mortality of patients with cirrhosis and septic shock was high. We found that inappropriate and delayed appropriate initial empiric antimicrobials were associated with significant increase in mortality. We also found that the empiric use of a single appropriate drug (compared with combination therapy with two or more antibiotics active for proven or suspected pathogens) for bacterial septic shock was associated with a significant increase in mortality. These findings were consistent among various clinically relevant subgroups.

Our study describes some of the unique features of septic shock in patients with cirrhosis. We found that patients with cirrhosis and septic shock were younger and had higher APACHE II scores compared with the general cohort of patients described.^21–23^ Similarly, body temperature at the time of presentation with septic shock was lower.^21–23^ Spontaneous bacterial peritonitis was present in 17.6% of the patients at the time of onset of septic shock. *Escherichia coli* and *Staphylococcus aureus* were the most common bacterial pathogens, and a strikingly high number of fungal infections (9.3%) were found. The use of activated protein C (1.4%) and corticosteroids (30.2%) were relatively low compared with that in the general cohort of patients described.^21–23^

We found a significant increase in mortality with inappropriate initial antimicrobial use. This is consistent with the findings of Kumar et al. in a heterogeneous cohort of septic shock patients.^22^ Although a sizable amount of literature is available on this topic,^29^ we are unaware of other similar work exploring such associations in a cohort of patients with cirrhosis. The strength of this finding is that it was consistent across various subgroups, including patients with gram-positive and gram-negative infections, pneumonia, and intra-abdominal infections. As such, inappropriate initial empiric antimicrobial therapy appears to be a strong yet modifiable determinant of outcomes of septic shock in patients with cirrhosis.

Delay in the initial empiric administration of appropriate antimicrobials is also associated with higher mortality. Over the last two decades, a large body of knowledge has emerged showing that timely antimicrobial administration is associated with significant improvement in outcomes.^30^, ^31^ This has been demonstrated in severe sepsis/septic shock,^21^ pneumonia,^32^ meningitis,^33^ bacteremia, and fungemia.^31^ Timely administration of antibiotics is a measure of the quality of care for community-acquired pneumonia. However, similar data are not available for patients with cirrhosis and septic shock. Given the high mortality of this group of patients, it is entirely possible that earlier administration of appropriate antimicrobial therapy would have resulted in better outcomes.

The final significant finding is that the empiric use of a single appropriate antibiotic (monotherapy) is associated with increased mortality in bacterial septic shock in this cohort. To our knowledge, no previous studies have addressed the exact question. A small study by McCormick et al.^34^ evaluated the efficacy and incidence of renal impairment with netilmicin plus mezlocillin compared with ceftazidime among 128 patients with cirrhosis and sepsis. Mortality rates were similar in the two groups. The reasons for our finding of a survival advantage with combination antibiotic therapy are not clear. This finding cannot be explained by a higher rate of coverage with combination therapy resulting from a high incidence of resistant bacteria in patients with cirrhosis who are often on prophylactic antibiotics. This is because combination therapy was defined as two or more antibiotics that were active for the isolated or suspected (in culture negative cases) pathogens. Additionally, the survival advantage was consistent in patients with or without multidrug-resistant organisms. We believe this is a novel finding that needs further exploration, given the high mortality and morbidity associated with septic shock in patients with cirrhosis.

Our data support a paradigm shift in the way we think about the natural progression of patients with cirrhosis. The natural course of cirrhosis has been considered to be irreversible and often fatal, except for patients who receive a liver transplant.^7^ Acute on chronic liver failure (ACLF) is a newly defined entity in which an acute insult in a previously compensated liver disease leads to deterioration and organ failure,^35^ which is partially reversible when identified early and patients receive early and appropriate intensive care support.^7^ A second principle in defining ACLF is the presence of an identifiable precipitating event, which in most cases is infection and sepsis.^7^ To improve outcomes in cirrhosis, early identification and management of these events is essential.^7^ We believe that our study contributes to this emerging field by guiding key aspects of antimicrobial therapy. Although the focus has been on therapies with unproven efficacy and safety profiles and low cost-effectiveness, such as liver support systems, the answer may be in redesigning the way we deliver routine care such as antimicrobial therapy to these patients.

Although the mortality of patients with cirrhosis who develop septic shock is very high, the diagnosis and treatment of this combination has been poorly studied. Part of the problem is the overlapping findings in sepsis and cirrhosis. Patients with cirrhosis have low baseline blood pressure, higher baseline heart rate, higher baseline breathing rate, and tend to not mount a vigorous febrile response.^18^ As such, identification of systemic inflammatory response syndrome in cirrhosis may prove difficult.^36^ Our study reflects this difficulty, as 25.5% of patients met the criteria for septic shock but were culture negative. Nevertheless, tests of interaction showed that the associations of appropriateness and timing of initial antimicrobial therapy and the use of single versus combination therapy were similar in culture-positive and culture-negative patients and in patients with documented or suspected infection. Furthermore, little progress has been made in the management of patients with cirrhosis and sepsis, as these patients tend to be excluded from studies of therapeutics in severe sepsis, such as the study of activated protein C in severe sepsis.^15^ Because the baseline central and mixed venous oxygen saturation tends to be higher in patients with cirrhosis,^18^ this specific goal for early goal directed therapy^16^ may not be applicable to these patients. Patients with cirrhosis are more prone to hypoglycemia and are not suitable candidates for intensive insulin therapy, either. Although antibiotics are commonly used in prophylaxis and treatment, the choice, timing, combinations and dosing have not been well studied. Our study addresses some of these points. The data suggest that appropriate and timely antimicrobial therapy and combination antibiotic therapy should be initiated before or, at the latest, concurrent with the onset of the hypotension of septic shock that typically happens several hours prior to ICU admission.

Possible explanations for the observed patterns of antimicrobial use may include process of care–related variations. Factors that influence prescription, transcription, preparation, dispensing and administration of antimicrobials among patients with cirrhosis and septic shock need to be investigated further as possible root causes. However, our study was not designed to delve into these issues.

Our study should be interpreted in light of its strengths and limitations. The strengths include the inclusion of patients from 28 ICUs based in three geographic regions. This lends the results of the study wide generalizability. To our knowledge, this is the first study specifically addressing the impact of various aspects of antibiotic use on outcomes among patients with cirrhosis and septic shock.

In terms of limitations, the results were not from a randomized controlled study. As such, the findings only highlight associations, and cause–effect relationships cannot be inferred. However, the combination and monotherapy groups, appropriate and inappropriate groups, and delay and early groups were generally comparable, and severity of illness as measured by APACHE II were not different. Observational studies such as ours are susceptible to confounding.^37^ Regression analysis is one way of adjusting for this in the statistical analysis.^37^ There are a number of possible factors that may influence outcome in acutely ill medical patients. Although we adjusted for severity of illness as measured by APACHE II and MELD scores, we cannot rule out residual confounding. However, the consistent and robust findings across various subgroups make it very unlikely that these findings are related to confounders alone. Furthermore, our classification of community-acquired and nosocomial infection followed the definitions used at the time of initiation of the database, and as such did not utilize the more recent concept of health care–associated infections that was introduced later.^38^ Nevertheless, we do not think this point affects the overall findings of the study, because the associations were observed in both groups.

In conclusion, this study shows that the inappropriate and delayed empiric antimicrobial therapy and single initial antibiotic therapy in patients with cirrhosis and septic shock is associated with significant increase in hospital mortality. Efforts need to focus on improving the choice and timing of empiric antibiotic therapy in this high-risk group.

## Appendix

Additional members of the CATSS Database Research Group include: Phillip Dellinger, M.D., Cooper Hospital/University Medical Center, Camden, NJ; Peter Dodek, M.D., St. Paul's Hospital, Vancouver, BC, Canada; Paul Ellis, M.D., University Health Network, Toronto, ON, Canada; Dave Gurka, M.D., Rush-Presbyterian-St. Luke's Medical Center, Chicago, IL; Jose Guzman, Harper Hospital, Detroit, MI; Sean Keenan, M.D., Royal Columbian Hospital, New Westminster, BC, Canada; Andreas Kramer, M.D., Brandon General Hospital, Brandon, MB, Canada; Aseem Kumar, Laurentian University, Sudbury, ON, Canada; Denny Laporta, M.D., Jewish General Hospital, Montreal, QC, Canada; Kevin Laupland, M.D., Foothills Hospital, Calgary, AB, Canada; Bruce Light, M.D., Winnipeg Regional Health Authority, Winnipeg, MB, Canada; Dennis Maki, M.D., University of Wisconsin Hospital and Clinics, Madison, WI; John Marshall, M.D., St. Michael's Hospital, Toronto, ON, Canada; Greg Martinka, M.D., Richmond General Hospital, Richmond, BC, Canada; Yazdan Mirzanejad, M.D., Surrey Memorial Hospital, Surrey, BC, Canada; Gourang Patel, Pharm.D., Rush-Presbyterian-St. Luke's Medical Center, Chicago, IL; Charles Penner, M.D., Brandon General Hospital, Brandon, MB, Canada; Dan Roberts, M.D., Winnipeg Regional Health Authority, Winnipeg, MB, Canada; John Ronald, M.D., Nanaimo Regional Hospital, Nanaimo, BC, Canada; Dave Simon, M.D., Rush-Presbyterian-St. Luke's Medical Center, Chicago IL; Sat Sharma, M.D., Winnipeg Regional Health Authority, Winnipeg, MB, Canada; Yoanna Skrobik, M.D., Hôpital Maisonneuve Rosemont, Montreal, QC, Canada; Kenneth E. Wood, DO, University of Wisconsin Hospital and Clinics, Madison, WI; Sergio Zanotti, M.D., Cooper Hospital/University Medical Center, Camden, NJ.

Associate members of the CATSS Database Research Group include: Muhammed Wali Ahsan, M.D., Winnipeg, MB, Canada; Mozdeh Bahrainian, M.D., Madison, WI; Rob Bohmeier, University of Manitoba, Winnipeg, MB, Canada; Lindsey Carter, BA, Winnipeg, MB, Canada; Harris Chou, BSc, of British Columbia, Vancouver, BC, Canada; Sofia Delgra, R.N., King Abdulaziz Medical City, Riyadh, Saudi Arabia; Winnie Fu, University of British Columbia, Vancouver, BC, Canada; Catherine Gonzales, R.N., King Abdulaziz Medical City, Riyadh, Saudi Arabia; Harleena Gulati, M.D., University of Manitoba, Winnipeg, MB, Canada; Erica Halmarson, M.D., University of Manitoba, Winnipeg, MB, Canada; Ziaul Haque, M.D., Montreal, QC, Canada; Johanne Harvey, R.N., Hôpital Maisonneuve Rosemont, Montreal, QC, Canada; Farah Khan, M.D., Toronto, ON, Canada; Laura Kolesar, R.N., St. Boniface Hospital, Winnipeg, MB, Canada; Laura Kravetsky, M.D., University of Manitoba, Winnipeg, MB, Canada; Runjun Kumar, University of Toronto, Toronto, ON, Canada; Nasreen Merali, M.D., Winnipeg, MB, Canada; Sheri Muggaberg, University of Manitoba, Winnipeg, MB, Canada; Heidi Paulin, University of Toronto, Toronto, ON, Canada; Cheryl Peters, R.N., M.D., University of Manitoba, Winnipeg, MB, Canada; Jody Richards, Camosun College, Victoria, BC, Canada; Christa Schorr, R.N., Cooper Hospital/University Medical Center, Camden, NJ; Norrie Serrano, R.N., King Abdulaziz Medical City, Riyadh, Saudi Arabia; Mustafa Suleman, M.D., Concordia Hospital, Winnipeg, MB; Amrinder Singh, M.D., Winnipeg, MB, Canada; Katherine Sullivan, University of Manitoba, Winnipeg, MB, Canada; Robert Suppes, M.D., University of Manitoba, Winnipeg, MB, Canada; Leo Taiberg, M.D., Rush Medical College, Chicago IL; Ronny Tchokonte, M.D., Wayne State University Medical School, Detroit, MI; Omid Ahmadi Torshizi, M.D., Montreal, QC, Canada; Kym Wiebe, R.N., St. Boniface Hospital, Winnipeg, MB, Canada.

## Abbreviations

ACLF: acute on chronic liver failure
aOR: adjusted odds ratio
APACHE: acute physiology and chronic health evaluation
CATSS: Cooperative Antimicrobial Therapy of Septic Shock
CI: confidence interval
ICU: intensive care unit
MELD: model for end-stage liver disease
WBC: white blood cell.
