# Supplementary material for: Antimicrobial therapeutic determinants of outcomes from septic shock among patients with cirrhosis
Source: Hepatology. 2012 Dec 4;56(6):2305–15. doi: 10.1002/hep.25931 (PMC3556696; doi:10.1002/hep.25931)
Supplement: Supplementary file 1 [file hep0056-2305-SD1.doc]

**Online Supplement**

**A. Criteria for Organ Failure/Dysfunctional**

### Cardiovascular System Dysfunction

- SBP ≤ 90 mm Hg or drop of more than 40 mm from normal systolic pressure

##### Or

- MAP ≤ 65 mm Hg for at least one hour despite adequate fluid resuscitation (2 L saline equivalent)

Or

- Need for vasopressors

### Renal Dysfunction

- Elevation of normal baseline serum creatinine to > 1.5 X normal value

### Respiratory System Dysfunction

- Ventilation required (more than immediate for surgery)

### Hematologic Dysfunction

- Platelet count < 80,000/mm

### Metabolic Dysfunction

- lactate level > 3 mmol/L (1.5 times the upper limit of the normal)

###  Central Nervous System Dysfunction

- An acute alteration in mental status not attributable to sedation

###  Hepatic Dysfunction

- Elevation of normal baseline serum total bilirubin to ≥ 2 mg/dl [US] or 35 ****mol/L [SI] (unless due to primary biliary disease e.g. ascending cholangitis, cholecystitis)

**B. Specific Definitions of Infections**

**Documented infection:** Meeting full CDC definitions for infection.

**Suspected infection:** Meeting partial CDC definitions for infection.

**Ventilator associated pneumonia:** Modified from the American College of Chest Physicians criteria. New or progressive radiographic infiltrate developed in conjunction with one of the following: radiographic evidence of pulmonary abscess formation, histologic evidence of pneumonia in lung tissue, a positive blood or pleural fluid culture of a plausible pathogen, or two of the following: fever (>38° C), leukocytosis (leukocyte count >10 x 10^3^/ mm^3^), purulent tracheal aspirate. Blood and pleural fluid cultures could not be related to another source and both had to be obtained within 48 hrs before or after the clinical suspicion of VAP

**Community-acquired pneumonia:** modified from CDC Definitions for Surveillance in Long-term Care Facilities 1991. Both of the following:

1. New infiltrate on CXR
2. Two of the following signs and symptoms:
   1. new or increased cough
   2. new or increased sputum production
   3. fever < 38oC
   4. pleuritic chest pain
   5. new or increased physical findings on chest exam (rales, rhonchi, wheezes, bronchial breathing)
   6. one of following indications of increased difficulty in breathing: SOB, tachypnea >25/min, or worsening of mental or functional status

**Nosocomial infections:** modified definitions according to the Center of Disease Control and Prevention Criteria, 1988. No evidence that the infection was present or incubating at the time of hospital admission

**Primary bloodstream infection**

1. Recognized pathogen isolated from blood culture and pathogen is not related to infection at another site
2. One of the following: fever (>38° C), chills, or hypotension and any of following
   1. Common skin contaminant ( diphtheroids, *Bacillus* sp., *Propionibacterium* sp., coagulase-negative staphylococci, or micrococci) isolated from two blood cultures drawn on separate occasions and organism is not related to infection at another site
   2. Common skin contaminant isolated from blood culture from patient with intravascular access device and physician institutes appropriate antimicrobial therapy
   3. Positive antigen test on blood and organism is not related to infection at another site

**Secondary bloodstream infection:** when an organism isolated from blood culture is compatible with related infection at another site. Exceptions to this are intravascular device-associated bloodstream infections, all of which are classified as primary even if localized signs of infection are present at the access site

**Catheter-associated infection:** bacteremia associated with catheter colonization (>15 colonies on semiquantitative culture) or overt tunnel/soft tissue infection

**Urinary tract infection:** includes symptomatic urinary tract infection, asymptomatic bacteriuria, and other infections of the urinary tract

**Symptomatic urinary tract infection:** must meet one of the following criteria:

1. One of the following: fever (>38° C), urgency, frequency, dysuria, or suprapubic tenderness and a urine culture of > 10^5^ colonies/ ml urine with no more than two species of organisms
2. Two of the following: fever (> 38° C), urgency, frequency, dysuria, or suprapubic tenderness and any of the following:
   1. Dipstick test positive for leukocyte esterase and/ or nitrate
   2. Pyuria (>10 white blood cells [WBC]/ ml^3^ or > 3 WBC/ high-power field of unspun urine)
   3. Organisms seen on Gram stain of unspun urine
   4. Two urine cultures with repeated isolation of the same uropathogen (Gram-negative bacteria or *Staphyloccus saprophyticus*) with > 10^2^ colonies/ml urine in nonvoided specimens
   5. Urine culture with < 10^5^ colonies/ml urine of single uropathogen in patient being treated with appropriate antimicrobial therapy
   6. Physician’s diagnosis (documented in patient’s chart)

**Central nervous system infection:** includes intracranial infection, meningitis or ventriculitis, and spinal abscess without meningitis

**Intracranial infection** (brain abscess, subdural or epidural infection, and encephalitis) must meet one of the following criteria:

1. Organism isolated form culture of brain tissue or dura
2. Abscess or evidence of intracranial infection seen during surgery or by histopathologic examination
3. Two of the following with no other recognized cause: headache, dizziness, fever (> 38° C), localizing neurologic signs, changing level of consciousness, or confusion, and physician institutes appropriate antimicrobial therapy if diagnosis is made antemotem and any of the following:
   1. Organism seen on microscopic examination of brain or abscess tissue obtained by needle aspiration or by biopsy during surgery or autopsy
   2. Positive antigen test on blood or urine
   3. Radiographic evidence of infection
   4. Diagnostic single antibody titer (IgM) or fourfold increase in paired serum samples (IgG) for pathogen

**Meningitis or ventriculitis:** must meet one of the following criteria:

1. Organism isolated from culture of cerebrospinal fluid (CSF)
2. One of the following with no other recognized cause: fever (> 38° C), headache, stiff neck, meningeal signs, cranial nerve signs, or irritability, and physician institutes appropriate antimicrobial therapy if diagnosis is made antemortem and any of the following:
   1. Increased white cells, elevated protein, and/ or decreased glucose in CSF
   2. Organism seen on Gram stain of CSF
   3. Organism isolated form blood culture
   4. Positive antigen test on CSF, blood, or urine
   5. Diagnostic single antibody titer (IgM) or fourfold increase in paired serum samples (IgG) for pathogen

**Gastrointestinal system infection:** include gastroenteritis, necrotizing enterocolitis, gastrointestinal tract infections, and intraabdominal infections

**Gastroenteritis** must meet either of the following criteria:

1. Acute onset of diarrhea (liquid stools for more than 12 hours) with or without vomiting or fever (> 38° C) and no likely noninfectious cause (e.g., diagnostic tests, therapeutic regimen, acute exacerbation of a chronic condition, psychologic stress)
2. Two of the following with no other recognized cause: nausea, vomiting, abdominal pain, or headache and any of the following:
   1. Enteric pathogen isolated from stool culture or rectal swab
   2. Enteric pathogen detected by routine or electron microscopy examination
   3. Enteric pathogen detected by antigen or antibody assay on feces or blood
   4. Evidence of enteric pathogen detected by cytopathic changes in tissue culture (toxin assay)
   5. Diagnostic single antibody titer (IgM)

**Gastrointestinal (GI) tract infections** (esophagus, stomach, small bowel, large bowel, and rectum), excluding gastroenteritis and appendicitis, must meet either of the following criteria:

1. Abscess or other evidence of infection seen during surgery or by histopathologic examination
2. Two of the following with no other recognized cause and compatible with infection of the organ or tissue involved: fever (> 38° C), nausea, vomiting, abdominal pain or tenderness and any of the following:
   1. Organism isolated form culture of drainage or tissue obtained during surgery or endoscopy or from surgically placed drain
   2. Organisms seen on Gram or KOH stain or multinucleated giant cells seen on microscopic examination of drainage or tissue obtained during surgery or endoscopy or from surgically place drain
   3. Organism isolated from blood culture
   4. Radiographic evidence of infection
   5. Pathologic findings on endoscopic examination (e.g., *Candida* esspohagitis or proctitis)

**Intraabdominal infection** (including gallbladder, bile ducts, liver [other than viral hepatitis], spleen, pancreas, peritoneum, subphrenic or subdiaphragmatic space, or other intraabdominal tissue or area not specified elsewhere) must meet one of the following criteria:

1. Organism isolated from culture of purulent material from intraabdominal space obtained during surgery or needle aspiration
2. Abscess or other evidence of intraabdominal infection seen during surgery or by histopathologic examination
3. Two of the following with no other recognized cause: fever (> 38° C), nausea, vomiting, abdominal pain or jaundice and any of the following:

a. Organism isolated from culture of drainage from surgically placed drain (e.g., closed suction drainage system, open drain, or T-tube drain)

- 1. Organisms seen on Gram stain of drainage or tissue obtained during surgery or needle aspiration
  2. Organism isolated from blood culture and radiographic evidence of infection

**Bacterial Peritonitis:** Bowel perforation, bowel ischemia, appendicitis

**Spontaneous Bacterial Peritonitis:** WBC > 500/uL or PMN > 250/uL or positive peritoneal fluid or blood culture of pathogenic organism

**Cholecystitis**: consistent clinical syndrome with radiologic support; operative support

**Ascending Cholangitis:** consistent clinical syndrome with radiologic support

**Pancreatitis (infected):** necrotizing pancreatitis on imaging with purulent drainage

**Skin and soft tissue infection:** includes skin infection (other than incisional wound infection), soft tissue infection, decubitus ulcer infection, burn infection, breast abscess or mastitis

**Skin infection** must meet either of the following criteria:

1. Purulent drainage, pustules, vesicles, or boils
2. Two of the following at affected site: localized pain or tenderness, swelling, redness, or heat and any of the following:
   1. Organism isolated form culture of aspirate or drainage form affected site; if organism is normal skin flora, must be pure culture of single organism
   2. Organism isolated from blood culture
   3. Positive antigen test on infected tissue or blood
   4. Clinical diagnosis with response to antibiotic therapy
   5. Diagnostic single antibody titer (IgM) or fourfold increase in paired serum sample (IgG) for pathogen

**Soft tissue infection:** (necrotizing fascitis, infectious gangrene, necrotizing cellulites, infectious myositis, lymphadenitis, or lymphangitis) must meet one of the following criteria:

1. Organism isolated form culture of tissue or drainage form affected site
2. Purulent drainage form affected site
3. Abscess or other evidence of infection seen during surgery or by histopathologic examination
4. Two of the following at affected site: localized pain or tenderness, redness, swelling, or heat and any of the following
   1. Organism isolated form blood culture
   2. Positive antigen test on blood or urine
   3. Diagnostic single antibody titer (IgM) or fourfold increase in paired serum samples (IgG) for pathogen

**Decubitus ulcer infection**, including both superficial and deep infection, must meet the following criterion: Two of the following: redness, tenderness, or swelling of wound edges and either of the following:

1. Organism isolated form culture of fluid obtained by needle aspiration or biopsy of tissue obtained from ulcer margin
2. Organism isolated from blood culture

**Burn infection** must meet one of the following criteria:

1. Change in burn wound appearance or character, such as rapid eschar separation, or dark brown, black, or violaceous doscoloration of the eschar, or edema at wound margin, and histologic examination of burn biopsy specimen that shows invasion of organisms into adjacent viable tissue
2. Change in burn wound appearance or character, such as rapid eschar separaration, or dark brown, black, or violaceous discoloration of the eschar, or edema at wound margin and either of the following:
   1. Organism isolated form blood culture in absence of other identifiable infection
   2. Isolation of herpes simplex virus, histologic identification of inclusions by light or electron microscopy, or visualization of viral particles by electron microscopy in biopsy specimens or lesion scrapings
3. Burn patient has tow of the following: fever (> 38° C) or hypothermia (< 36° C), hypotension (systolic pressure < 90mm Hg), oligura (< 20 mg/ hr), hyperglycemia at previously tolerated level of dietary carbohydrate, or mental confusion and any of the following:
   1. Histologic examination of burn biopsy specimen that shows invasion of organisms into adjacent viable tissue
   2. Organism isolated from blood culture
   3. Isolation of herpes simplex virus, histologic identification of inclusions by light or electron microscopy, or visualization of viral particles by electron microscopy in biopsy specimens or lesion scrapings

**Breast abscess** or mastitis must meet one of the following criteria:

1. Organism isolated form culture of affected breast tissue or fluid obtained by incision and drainage or needle aspiration
2. Breast abscess or other evidence of infection seen during surgery or by histopathologic examination
3. Fever (> 38° C), local inflammation of the breast, and physician’s diagnosis

**Surgical wound infection:**  includes incisional surgical wound infection and deep surgical wound infection

**Incisional surgical wound infection:** must meet the following criterion: Infection occurs at incision site within 30 days after surgery and involves skin, subcutaneous tissue, or muscle located above the fascial layer and any of the following:

1. Purulent drainage from incision of drain located above fascial layer
2. Organism isolated form culture of fluid from wound closed primarily
3. Surgeon deliberately opens wound, unless wound is culture-negative
4. Surgeon’s or attending physician’s diagnosis of infection (documented in the patient’s chart)

**Deep surgical wound infection:** must meet the following criterion: Infection occurs at operative site within 30 days after surgery if no implant is left in place or within 1 year if an implant is in place and infection appears related to surgery and infection involves tissues or spaces at or beneath fascial layer and any of the following:

1. Purulent drainage from drain placed beneath fascial layer
2. Wound spontaneously dehisces or is deliberately opened by surgeon when patient has fever (> 38° C) and/ or localized pain or tenderness, unless wound is culture-negative
3. An abscess or other evidence of infection seen on direct examination, during surgery, or by histopathologic examination
4. Surgeon’s diagnosis of infection (documented in the patient’s chart)

Reference

1. Kollef MH, Sherman G, Ward S, Fraser VJ. Inadequate antimicrobial treatment of infections: a risk factor for hospital mortality among critically ill patients. *Chest* 1999;115:462-474.
2. Garner JS, Jarvis WR, Emori TG, Horan TC, Hughes JM. CDC definitions for nosocomial infections. *Am J Infect Control* 1988;16:128-140.
3. Knaus WA, Sun X, Nystrom PO, Wagner DP. Evaluation of definitions for sepsis. *Chest* 1992;101:1656-1652.
4. McGeer A, Campbell B, Emori TG, et al. Definitions of Infection for Surveillance in Long-term care facilities. Am J Infect Control 1991;1:1-7

**C. Rules to Assign Clinical Significance to Microbial Isolates**

1. Clinically significant isolates from either local site and/or blood cultures were required to have been obtained within 48 h of the onset of shock.
2. The following were considered to represent clinically significant isolates:
   1. A blood culture positive for any pathogen other than coagulase-negative staphylococci or other skin contaminants;
   2. Any growth from a normally sterile site (*e.g.*, gall bladder, bronchial lavage, peritoneal, pleural fluid, or operative tissue specimen) apart from coagulase-negative staphylococci and other skin contaminants;
   3. Growth of a pathogen in a sputum sample from a patient with respiratory signs and symptoms, or a new infiltrate seen on chest radiography, with no other likely source of infection;
   4. Growth of a pathogen in a urine sample (> 10^8^ organisms per liter) with either local clinical symptoms or in the absence of a more plausible clinical infection site;
   5. Growth from a deep biopsy specimen or a deep aspirate of a finding in soft tissue or skin;
   6. Concurrent congruent positive semi-quantitative catheter colonization (> 15 colonies) with blood culture or clinical evidence of site infection; and
   7. A positive direct measurement of *Legionella pneumophila* antigen in the urine; or *S pneumoniae*, *Neisseria meningitides*, or *Haemophilus influenzae* in the sputum.
3. Candida lung isolates were considered to be colonizers unless also isolated from multiple other normally sterile sites in which case disseminated infection was diagnosed. Enterococci were considered to be clinically significant only in the absence of other more plausible pathogens.
4. *Staphylococcus epidermidis* was uniformly considered to be incapable of causing septic shock. Other coagulase-negative staphylococci were similarly considered to be unlikely to cause septic shock unless present as a sole isolate in multiple blood cultures in the absence of evidence of endovascular infection.

**D. Designation of Appropriateness of Antimicrobial Therapy**

1. The following were considered appropriate therapy even in the absence of specific sensitivity testing: (a) group A, B, and G Streptococcus treated with all β-lactams; (b) all Gram-positive bacteria except enterococci treated with vancomycin; (c) anaerobes treated with metronidazole, β-lactam inhibitor combinations, and carbapenems; and (d) organisms treated with β-lactamase inhibitor combinations if treated with the β-lactam alone.
2. The following were considered inappropriate therapy even in absence of specific sensitivity testing: (a) Enterococci treated with all cephalosporins and trimethoprim/sulfamethoxazole; (b) *Enterococcus faecalis* sensitive to quinupristin-dalfopristin; and (c) any bacteria treated with monotherapy with aminoglycoside at standard dosing every 8 h.
3. Legionella species were considered appropriately treated with macrolides or quinolones.
4. Treatment with oral or IV metronidazole or oral vancomycin along with broad-spectrum antienteric antimicrobial therapy was considered to be a requirement for appropriate antimicrobial therapy of septic shock caused by *Clostridium difficile* entercolitis.
5. Clindamycin, macrolides, and third-generation cephalosporins were not considered appropriate for the treatment of *S aureus* infection irrespective of listed sensitivity.
6. Cefotaxime and ceftriaxone were not considered appropriate therapy for

Pseudomonas aeruginosa infection irrespective of listed sensitivity.

1. In cases where multiple isolates were found at a local site, appropriate therapy was considered to have been delivered if the densest pathogen was covered. If multiple pathogens were isolated at a similar density, all pathogens were required to have been covered.
2. For multiple simultaneous blood isolates, appropriate therapy had to cover all

pathogens.

E. Tables

Online Table 1: Results showing p value for interaction of the subgroups with the appropriateness, timing and combination of antimicrobial therapy.

|  | Inappropriate antimicrobial  therapy | Delay in effective antimicrobial therapy | Using single inappropriate antimicrobial therapy |
| --- | --- | --- | --- |
|  | **P** | **P** | **P** |
| Documented vs. suspected infection | 0.96 | 0.65 | * |
| Culture-positive vs. culture-negative | 0.96 | 0.32 | * |
| Bacteremia vs. no bacteremia | 0.22 | 0.26 | 0.59 |
| Community-acquired vs. nosocomial infection | 0.32 | 0.02 | 0.65 |
| Pneumonia vs. no pneumonia | 0.23 | 0.86 | 0.42 |
| Intraabdominal infection vs. no intrabdominal infection | 0.40 | 0.53 | 0.1 |
| Immunocompromised vs. non-immunocompromised | 0.72 | 0.62 | 0.64 |
| Country (Canada, USA and Saudi Arabia) | 0.95 | 0.42 | 0.45 |
| Study periods (four periods) | 0.24 | 0.08 | 0.36 |

Online Table 2: Differences among the three study countries in appropriateness, timing and combination of antimicrobial therapy.

|  | **Canada (n = 461)** | **USA (n = 61)** | **Saudi Arabia**  **(n = 113)** | **P** |
| --- | --- | --- | --- | --- |
| **Appropriateness of initial antimicrobial therapy** | | | | |
| Inappropriate | 118 (25.6) | 17 (27.9) | 20 (17.7) | 0.17 |
| Appropriate | 343 (74.4) | 44 (72.1) | 93 (82.3) |
| **Timing of first appropriate antibiotic** | | | | |
| Prior to hypotension onset | 79 (18.5) | 13 (22.8) | 21 (20.2) | 0.71 |
| After hypotension onset | 349 (81.5) | 44 (77.2) | 83 (79.8) |
| Timing of antimicrobial treatment,  mean ± SD | 17.1 ± 23.3 | 16.2 ± 29.0 | 12.7± 19.6 | 0.31 |
| **Appropriate antimicrobial therapy during the course of shock** | | | | |
| Who received single therapy | 168 (74.7) | 12 (50) | 46 (75.4) | 0.03 |
| Who received combination therapy | 57 (25.3) | 12 (50) | 15 (24.6) |
